# Effect of the Universal Health Coverage Healthcare System on Stock Returns During COVID-19: Evidence From Global Stock Indices

**DOI:** 10.3389/fpubh.2022.919379

**Published:** 2022-07-12

**Authors:** Chia-Hsien Tang, Yen-Hsien Lee, Win Liu, Li Wei

**Affiliations:** ^1^College of Accounting and Auditing, Guangxi University of Finance and Economics, Guangxi Accounting Research Institution, Nanning, China; ^2^Department of Finance, Chung Yuan Christian University, Taoyuan, Taiwan

**Keywords:** coronavirus disease (COVID-19), abnormal return, universal health coverage, total confirmed cases, global stock market

## Abstract

The increased uncertainty caused by a sudden epidemic disease has had an impact on the global financial market. We aimed to assess the primary healthcare system of universal health coverage (UHC) during the coronavirus disease (COVID-19) pandemic and its relationship with the financial market. To this end, we employed the abnormal returns of 68 countries from January 2, 2019, to December 31, 2020, to test the impact of the COVID-19 outbreak on abnormal returns in the stock market and determine how a country's UHC changes the impact of a sudden pandemic on abnormal returns. Our findings show that the sudden onset of an epidemic disease results in unevenly distributed medical system resources, consequently diminishing the impact of UHC on abnormal returns.

## Introduction

The first death due to the coronavirus disease (COVID-19) occurred on January 11, 2020, in China. Subsequently, the COVID-19 outbreak spread rapidly worldwide in early 2020. Thailand reported a confirmed case on January 13, 2020, the first case outside China. On August 5, 2021, the largest proportionate increase in new cases was reported by the Americas (14%) and the Western Pacific Region (19%), with 1.3 million and over 375,000 new cases reported, respectively (1). The recent delta variant of COVID-19 is creating global concern, highlighting the risks faced by people who do not have access to primary healthcare *via* universal health coverage (UHC). This system enables everyone to obtain fair access to health services, including prevention and treatment, with no extra cost incurred, especially during the COVID-19 crisis period.

The spread of COVID-19 in over 190 countries has prompted an in-depth look at the various health effects and responses to COVID-19 in the marketplace due to long-ignored global health risks. Every country has begun focusing on the defense against novel viruses by providing full access to the medical system during the COVID-19 outbreak (2). Strong health systems, based on primary health care and UHC, are the foundation for an effective response to COVID-19. Specifically, where health coverage is linked to employment, an economic shock that leads to a loss of formal sector jobs also has negative consequences for the financial market. Therefore, in countries that have historically relied on contributory, employment-linked coverage, it is essential to inject general budget revenues into the system to reduce the system's vulnerability to job losses and ensure that the essential actions needed to respond to COVID-19 can be implemented.

Regarding panic selling, the sudden, large-scale sale of securities caused a significant decline in stock prices in the short term. The COVID-19 outbreak has also directed scholarly attention to exploring the impact of macroeconomic factors on stock returns, especially in the short term. Goodell and Huynh (3) and Shahzad et al. (4) suggest that the COVID-19 outbreak had a major impact on the financial market, which has been identified as having negative abnormal returns. Additionally, many scholars have empirically shown a link between major events and stock returns. In turn, they have revealed stock price fluctuations related to a specific event, such as an election (5), terrorist attacks (6), and disease outbreaks, specificallyanimal diseases, severe acute respiratory syndrome (SARS), and Ebola (7–9). He et al. (10), Alam et al. (11), and Mazur et al. (12) showed that the United States, Australia, and China all had negative abnormal returns in the stock market due to the COVID-19 outbreak. Liu et al. (13), Alali (14), and Singh et al. (15) examined multiple severely infected countries, finding that COVID-19 caused abnormal returns, not only in the examined country but also influencing numerous others.

Dongarwar and Salihu (16) reported that the COVID-19 death rate in a country with UHC is twice as low as that in a country without UHC. Apergis and Apergis (17) and Song et al. (18) used the growth rate of confirmed COVID-19 cases as a proxy and found that when the number of confirmed cases increased, the market index in China and America decreased. Ashraf (19) and Khan et al. (20) proved that an increase in the number of confirmed cases also affects the market index of each country. Concurrently, the worldwide rate of unexpected confirmed COVID-19 cases is increasing; the Centers for Disease Control and Prevention (CDC) reported that the infection and transmission rates of COVID-19 are much higher than expected. An influenza carrier can infect up to 1.3 people, while a COVID-19 carrier can infect 5–6 people (21). As a result of the increased number of confirmed COVID-19 cases, medical resources will be unevenly distributed, which will eventually affect the stock market.

This study aims to test the response of abnormal returns to a sudden pandemic disease and how the national UHC of a country changes the impact of sudden pandemic diseases on abnormal returns. Unlike previous studies that only examined specific events and their effects on the stock market in the short term, our study considers the UHC's effects on the stock market in relation to the COVID-19 outbreak in the long term. Consistent with the studies of Goodell and Huynh (3) and Shahzad et al. (4), the results suggest that the COVID-19 outbreak had a major impact on the financial market. This fills a gap in the current literature by providing an empirical framework demonstrating the healthcare system's connection to a sudden pandemic disease and its effects on the stock market in the long term.

The remainder of this paper is organized as follows. Section 2 discusses the Relevant Literature, and Section 3 presents the Data and the Methodology used. Section 4 discusses the Empirical Results of the findings. Finally, Section 5 concludes the study.

## Relevant Literature

### The Correlation Between the COVID-19 Outbreak and Abnormal Returns

Efficient market theory states that stock prices reflect all information, and consistent alpha generation is impossible. The sensitivity of any information and unexpected events in the stock market will eventually reflect or force stock prices upward or downward. Since the COVID-19 outbreak was confirmed in 2019, various stock market indices have collapsed and intensified worldwide (10, 22, 23). It was revealed that, after China officially notified the WHO of the epidemic's outbreak on January 23, 2020, both Shanghai A shares and Shenzhen A shares had negative abnormal stock returns, especially in the transportation, mining, entertainment, and tourism industries. In addition, similar results were found after the first confirmed case was discovered in Australia on February 27, 2020, and after the announcement of COVID-19 as a global pandemic in the United States on March 11, 2020 (11, 12).

Alali (14) examined the top five Asian stock market indices (Shanghai Composite Index, Nikkei 225, Mumbai Sensitive 30 Index, Hang Seng Index, and South Korea Composite Stock Index) to test their reaction to the WHO's announcement of COVID-19 as a global pandemic. The empirical results show that the announcements have a significantly negative relationship with the cumulative abnormal returns in all stock market indices. In addition, Heyden and Heyden (24) and Bash (25) studied Europe and the United States and the top 30 countries most severely impacted by confirmed cases of COVID-19, finding negative abnormal returns in the stock markets. Moreover, Liu et al. (13) studied 21 significantly infected countries, and Singh et al. (15) studied 20 badly affected countries and found that the pandemic had a negative impact on their respective stock markets and generated negative abnormal returns.

Recently, Pandey and Kumari (26) collected 49 stock indices from both developed and emerging markets worldwide and found significant negative abnormal returns on global stock markets after the WHO declared COVID-19 a public health emergency of international concern. Among these, Asian stock markets fared the worst among the 49 stock indices.

Prior studies indicate that a sudden pandemic disease is followed by negative abnormal returns in a certain country or region (5, 6, 8, 9). Bouri et al. (27) that assets' connectedness of returns varied before and after the COVID-19 outbreak. Similarly, negative abnormal returns occurred in each country after its first confirmed case was reported, and when the WHO declared COVID-19 a global pandemic on March 11, 2020 (10–12, 14). Consequently, this study considers the COVID-19 outbreak a global pandemic, confirmed by the WHO, which eventually spread globally and generated negative abnormal returns in stock markets worldwide. Thus, we construct Hypothesis 1 as follows:

**Hypothesis 1:** The spread of the COVID-19 outbreak worldwide generates negative abnormal returns in global stock markets.

### The Correlation of Abnormal Returns With COVID-19 and Universal Health Coverage

Broad coverage from a good healthcare system improves health indicators, reduces health inequalities, and enhances economic development. The COVID-19 outbreak has amplified the progress of the establishment of strong and resilient health care systems. A recent study by 16 indicated that countries with UHC had a lower number of confirmed COVID-19 cases. The study by Djilali et al. (28) has evidently shown that vaccination is a strategy to limit the spread of the COVID-19 disease; the high number of vaccination rates can decrease the infection and fatality rates, as does UHC. Additionally, the World Health Organization (2) announced that UHC allows governments to effectively address hazards caused by COVID-19 worldwide, either directly or indirectly. Such a significant market response is not mirrored in the instance of a reduction in cases. Empirical evidence by Benjamin (29), McKibbin and Fernando (30), Banik et al. (31), and Bentout et al. (32) show that a robust and resilient healthcare system helps mitigate the exposure risk to COVID-19. This is because a healthcare system has a greater positive impact on the spread of the virus in less developed, high population-density countries and decreases fatality rates in countries with high infection rates. The healthcare system is consistent with a statement from Bill Gates, co-chair of the Bill & Melinda Gates Foundation, who said that a multi-specialty Global Epidemic Response and Mobilization (GERM) team helps to strengthen health systems in an effort to build a resilient system that will help reduce the damage of the next pandemic. So, the capacity to produce billions of vaccines has been initiated; the funding to pay for them and the systems to deliver them everywhere are vital to the global fight against the pandemic.

Additionally, recent studies have revealed that a well-organized healthcare system can mitigate the negative outcomes of COVID-19 (29–31). Few previous studies have investigated the impact of UHC on the stock market or tested the correlation effect; hence, this study develops the following hypothesis:

**Hypothesis 2:** UHC is positively correlated with abnormal returns.

### The Effect of a High Rate of Confirmed COVID-19 Cases on UHC and Its Link to Abnormal Returns

When an epidemic occurs, the basic healthcare system is disrupted or damaged by the sudden influx of numerous patients. Therefore, medical treatment no longer fulfills the needs of society or individuals to maintain their daily lives. Chaos and panic ensue because of insufficient or limited resources. Liu et al. (13) stated that an increase in confirmed COVID-19 cases enhances investors' pessimistic emotions toward the stock market and creates market uncertainty, which, in turn, affects stock prices and generates negative abnormal returns. A study by Ashraf (19) involving 64 countries also showed that the impact of the number of confirmed cases on stock prices was greater than that of the number of deaths. Once the number of confirmed COVID-19 cases showed an upward trend, the volatility of stock prices followed a downward trend. Khan et al. (20) also discovered that the growth rate of new weekly diagnoses was significantly negatively correlated with stock prices. As the number of new diagnoses rises by 1% in a week, stock market returns fall by 0.24%.

Apergis and Apergis (17) and Saif-Alyousfi (33) studied the increase in confirmed cases or deaths as proxy variables for COVID-19; they found that an increase in confirmed cases or mortalities was significantly negatively correlated with stock returns in China. Moreover, Song et al. (18) reported that an increase in diagnoses was significantly negatively correlated with stock returns in the United States, especially in the catering industry. Furthermore, Pandey and Kumari (26) found that the total number of confirmed cases and fatalities has a negative impact on cumulative abnormal returns in developed and emerging markets.

With the increased number of confirmed COVID-19 cases, UHC as the primary healthcare system will, due to a sudden and large increase in the number of patients, eventually affect the stock market negatively. The increase in the number of confirmed COVID-19 cases is based on the theory of insufficient resource allocation. The public will panic and cause the collapse of the medical system, and the UHC function may also be weakened by abnormal returns. Thus, a strategy of either the government issuing public interventions, such as a lockdown (33), or a full coverage of vaccines for people is needed to reduce the epidemic damage that could be brought by the serious COVID-19 outbreak as well as to stop the virus spreading to others (34, 35). Based on the results of prior studies, we expect an increase in the diagnoses of COVID-19 to overwhelm the UHC and weaken its positive impact; therefore, the study constructs the following hypothesis:

**Hypothesis 3:** A high number of COVID-19 diagnoses will affect the impact of UHC on abnormal returns.

## Data and Methodology

### Data

This study collected major global stock indices divided by region, namely Asia, Europe, America, Africa, and Oceania, as shown in Table 1. The stock market index in Europe was weighted as 35%. Asia, America, Africa, and Oceania are weighted as 33, 14, 16, and 2%, respectively, excluding those countries whose data were not fully available^1^. Table 1 represents the stock indices in 68 countries, and it was decided to use these indices to investigate the influence of the COVID-19 outbreak.

**Table 1:** Major stock market indices.

**Country/area**	**Stock index**	**Country/area**	**Stock index**
Austria	ATX	Netherlands	AEX
Australia	S and P ASX 200	Norway	OSE Benchmark
Argentina	S and P Merval	New Zealand	NZX 50
Belgium	BEL 20	Namibia	FTSE NSX Overall
Bulgaria	BSE SOFIX	Nigeria	NSE 30
Brazil	Bovespa	Poland	WIG 30
Bangladesh	DSE 30	Portugal	PSI 20
Croatia	CROBEX	Peru	SandP Lima General
Canada	S and P TSX Composite	Pakistan	Karachi 100
Chile	S and P CLX IPSA	Philippines	PSEi Composite
Colombia	COLCAP	Qatar	QE General
China	Shanghai Composite	Romania	BET
Denmark	OMX Copenhagen 20	Russia	MOEX
Ecuador	Guayaquil select	Serbia	Belex 15
Egypt	EGX 70 EWI	Slovakia	SAX
France	CAC 40	Slovenia	Blue-Chip SBITOP
Germany	DAX	Spain	IBEX 35
Greece	AGC	Sweden	OMX Stockholm 30
Hungary	Budapest SE	Switzerland	SMI
Iceland	ICEX Main	South Korea	KOSP
Ireland	ISEQ Overall	Saudi Arabia	Tadawul All Share
Italy	FTSE MIB	Singapore	FTSE STS
India	BSE Sensex 30	Sri Lanka	CSE All-Share
Indonesia	Jakarta SEC	South Africa	Top 40
Iraq	ISX Main 60	Thailand	SET Index
Israel	TA 35	Turkey	BIST 100
Japan	Nikkei 225	Tanzania	All Share
Jamaica	JSE Market	Tunisia	Tunindex
Kazakhstan	KASE	Ukraine	PFTS
Kenya	NSE 20	United Kingdom	FTSE 100
Lebanon	BLOM Stock	United States	SandP 500
Mexico	SandP BMV IPC	UAE	ADX General
Malaysia	FTSE KLCI	Vietnam	VN
Morocco	Moroccan All Shares	Zambia	LSE All Share

*FTSE STS, FTSE Straits Times Singapore; AGC, Athens General Composite; UAE, United Arab Emirates*.

The study employed a market model to calculate abnormal returns in each region. Therefore, the MSCI All-Country World Equity Index, an international benchmark index representing global market performance, was used to calculate the abnormal returns of all the stock markets listed in Table 1. Daily closing prices were collected from the website investing.com, which offers free historical data from January 2, 2019, to December 31, 2020, in all regions' indices.

### Methodology

In this study, we adopted event study method to evaluate stock reaction to a specific event. Thus, in order to precisely capture the effect of UHC during the COVID-19 outbreak, we have adopted a short-term (5 days), mid-term (10–60 days), and long-term (180 days) to test stock movement. By doing so, this study is aiming to precisely capture the volatility of stock abnormal returns during COIVD-19 outbreak.

#### Universal Health Coverage Definition

Universal health coverage service coverage index, established by the WHO, aims to ensure that people receive adequate healthcare without an undue burden on their finances. This study uses the WHO UHC database to collect UHC data for 68 countries. The 34 provides the following explanation for UHC:

“The goal of universal health coverage is threefold:

Equity in access: everyone who needs health services should receive them, not only those who can pay for services.
Sufficient quality: health services should be of sufficient quality to improve the health of those receiving the services.
No undue financial risk: the cost of using health services should not put people at risk of financial harm.” (p. 2)

**Figure 1:**
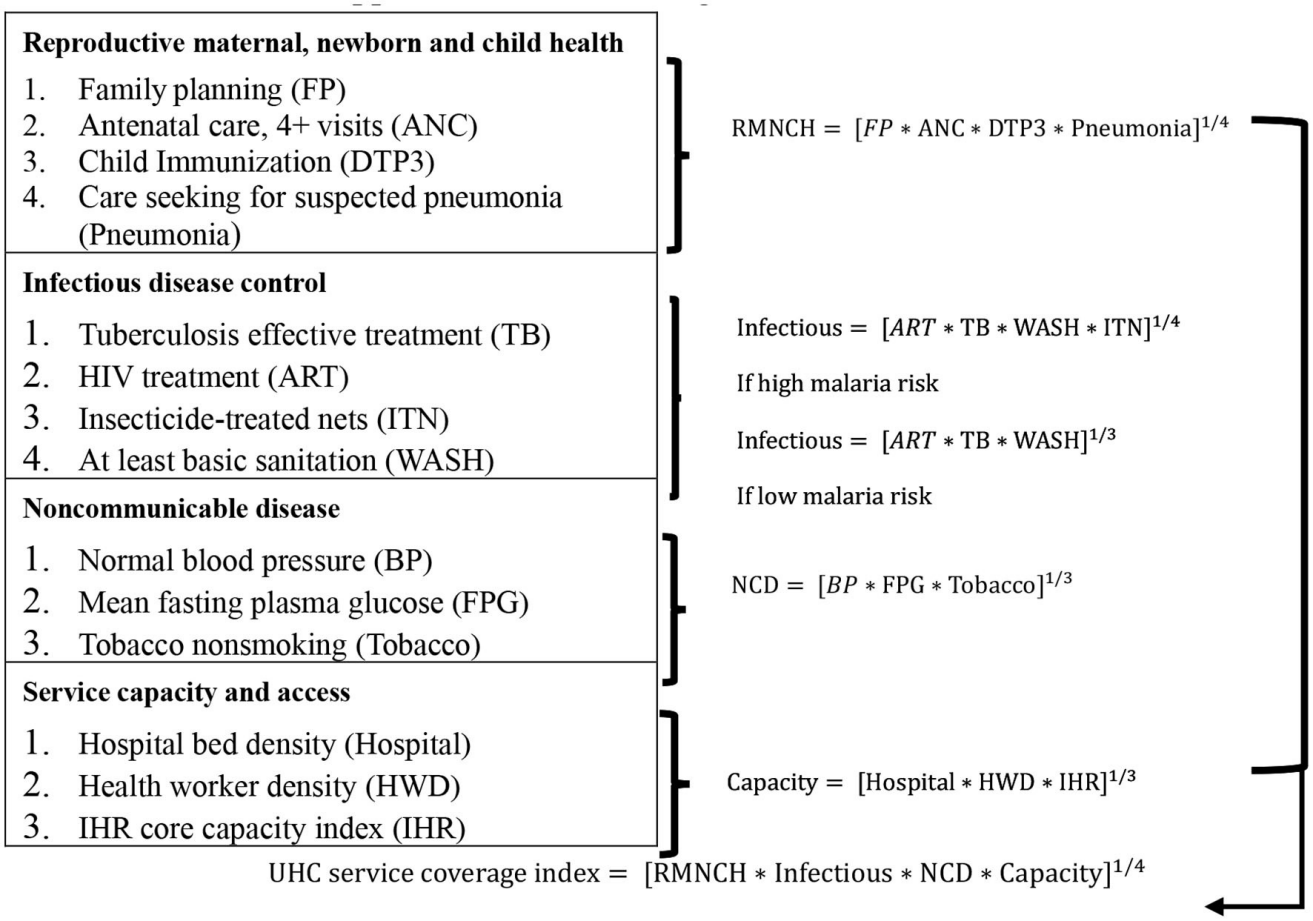
UHC service coverage index (2)^2^.

The UHC calculation approach is shown in Figure 1.

#### Event Study

Many methodologies have been used to model event studies to evaluate abnormal returns. However, the event study method used to examine the impact of COVID-19 on the volatilities of all affected countries is the most suitable for capturing stock price movements. Previous studies used event studies to test the impact of certain events, such as initial public offerings, seasoned equity offerings, and stock splits on a company's stock (36, 37). Nevertheless, in recent years, an increasing number of scholars have used the event study method to capture the impact of unexpected events, such as SARS, Ebola, and Middle East Respiratory Syndrome (MERS), on stock prices (8, 9).

Bash (25) and Heyden and Heyden (24) stated that a single event day in the market could not accurately capture the influence of COVID-19 on abnormal returns. Therefore, they used the first confirmed case in each country as an event day to test for abnormal returns. Nevertheless, Alali (14) found that, after the WHO's official declaration, stock prices fell sharply compared to that of 30 days before and after the report of the first confirmed case in each country. To precisely capture the impact of stock movement on COVID-19, this study adopted the official WHO declaration of COVID-19 as a global pandemic on March 11, 2020, as an event day to test the response of global stock markets.

### Measure of Returns and Hypothesis

#### Market Model of Abnormal Returns


(1) $$\begin{array}{r} AR_{i,t}=R_{i,t}-\left(\alpha_{0}+\alpha_{1}\times R_{m,t}\right) \end{array}$$

*AR*_*i, t*_ and *R*_*i, t*_ are the abnormal return and the real return of stock market *i* on day *t*, respectively; *R*_*m, t*_ is the market return of the MSCI all-country world equity index on day *t*, with α_0_ and α_1_ as the coefficients of the ordinary least squares (OLS) from the estimation period (−150, −1). In the stock market, the accurate event date or time of abnormal return is difficult to define; thus, a period of observation is needed to define the event date or time of abnormal return. Hence, cumulative abnormal return (CAR) is where stock market i from *t*_0_ to *t*_1_ is calculated based on Equation (2) to verify whether COVID-19 leads to negative abnormal returns:


(2) $$\begin{array}{r} CAR_{i}\left(t_{0},\;t_{1}\right)=\overset{t_{1}}{\underset{t=t_{0}}{\sum }}AR_{i,t} \end{array}$$

where *t*_1_ is defined as the event days of 5, 10, 30, 60, and 180.

#### Universal Health Coverage Impact on Abnormal Returns

This study mainly aims to examine the relationship between UHC and abnormal returns and whether there was a positive relationship between UHC and abnormal returns during the COVID-19 outbreak. Therefore, the study expects a positive (θ_1_ > 0) relationship between UHC and CAR by testing Equation (3), as follows:


(3) $$\begin{array}{r} CAR_{i}^{N}=\theta_{0}+\theta_{1}UHC_{i}+\beta X_{i}+\varepsilon_{i} \end{array}$$

$CAR_{i}^{N}$ is the CAR during a specific period before and after the event day with N for (0, 5), (0, 15), (0, 30), (0, 60), and (0, 180). *UHC*_*i*_ denotes that country i has UHC; *X*_*i*_ is defined as the control variables, such as log (gross domestic product), uncertainty avoidance index, net domestic credit divided by gross domestic product (GDP), log (population), political stability and no violence, and regulatory requirements. Log (GDP) and Credit/GDP are taken from the World Bank Open Data, and they measure the level of economic development (38). The uncertainty avoidance index is taken from the study by Hofstede et al. (39) on cross-country differences in national culture and measurement of the degree of investor uncertainty aversion.

Log (population) is taken from the World Bank Open Data and measures the size of each stock market (20). Both political stability and no violence and regulatory requirements, which also represent the quality of political safety, are taken from the World Bank's GOV Data 360 (20). These control variables jointly capture the cross-broader differences in stock market returns caused by country governance or macroeconomic differences between countries. ε_*i*_ is the residual.

#### Impact of High COVID-19 Infection Rates With UHC on Abnormal Returns

To verify the impact of high rates of COVID-19 infection on CARs, we modified the baseline regression to examine the indirect impact of UHC on abnormal returns as Equation (4) to investigate Hypothesis 3: whether the severity of COVID-19 weakens the positive impact of UHC on abnormal returns.


(4) $$\begin{array}{r} CAR_{i}^{N}=\theta_{0}+\theta_{1}UHC_{i}+\theta_{2}TC_{i}+\theta_{3}UHC_{i}\times TC_{i}+\beta X_{i}+\;\varepsilon_{i} \end{array}$$

where *TC*_*i*_ is the cumulative confirmed cases of stock market *i* from the date of diagnosis of the first patient in each country to that of the research event. Total confirmed cases are taken from the website *Our World in Data*, which discloses the number of fatalities and confirmed cases in all countries globally. The interaction term *UHC*_*i*_ × *TC*_*i*_ is a dummy variable that divides the total confirmed cases into three groups, which is lowest, medium, and largest, and then sets the highest equal to 1 and the others equal to 0. The main variable of θ_3_ denotes UHC reactions to abnormal returns in relation to the total number of confirmed COVID-19 cases. Thus, the expectation of the relationship between the interaction variable of θ_3_ and CAR is negative (θ_3_ < 0).

## Empirical Results

### Description of Samples

Table 2 presents the descriptive statistics of the stock market index variables for all the sample countries. This study analyzed a sample of 68 countries. The mean variable of stock market indices is either 0.00 or −0.00, which indicates that all sample countries had zero present returns in stock markets, which is consistent with the random walk property of stock market returns (19). In addition, the minimum and maximum values of all 68 countries ranged from −0.21 to 0.15, which indicates that all sample returns were from −21 to 15% during the COVID-19 outbreak. Among these, Argentina, Italy, and Brazil had the lowest stock returns of −0.21, −0.16, and −0.18, respectively.

**Table 2:** Descriptive statistics of major countries' stock market indices.

**Country**	**Stock index**	**Obs**	**Mean**	**SD**	**Min**	**Max**	**Country**	**Stock index**	**Obs**	**Mean**	**SD**	**Min**	**Max**
Argentina	S and P Merval	764	0.00	0.01	−0.21	0.04	Morocco	MAS	780	−0.00	0.01	−0.09	0.05
Australia	S and P ASX 200	798	0.00	0.01	−0.10	0.07	Namibia	FTSE NSX Overall	786	0.00	0.01	−0.04	0.04
Austria	ATX	790	−0.00	0.02	−0.15	0.10	Netherlands	AEX	805	0.00	0.01	−0.11	0.08
Bangladesh	DSE 30	727	−0.00	0.01	−0.06	0.10	NewZealand	NZX 50	918	0.00	0.01	−0.06	0.07
Belgium	BEL 20	805	−0.00	0.01	−0.15	0.07	Nigeria	NSE 30	782	−0.00	0.01	−0.06	0.06
Brazil	Bovespa	777	0.00	0.02	−0.16	0.13	Norway	OSE Benchmark	648	0.00	0.01	−0.09	0.05
Bulgaria	BSE SOFIX	777	−0.00	0.01	−0.11	0.04	Pakistan	Karachi 100	782	0.00	0.01	−0.07	0.05
Canada	S and P TSX Composite	790	0.00	0.01	−0.13	0.11	Peru	SandP Lima General	794	0.00	0.01	−0.11	0.05
Chile	S and P CLX IPSA	782	−0.00	0.01	−0.15	0.08	Philippines	PSEi Composite	769	−0.00	0.01	−0.14	0.07
China	Shanghai Composite	763	0.00	0.01	−0.08	0.06	Poland	WIG 30	784	−0.00	0.01	−0.14	0.06
Colombia	COLCAP	767	−0.00	0.01	−0.13	0.12	Portugal	PSI 20	805	−0.00	0.01	−0.10	0.08
Croatia	CROBEX	779	−0.00	0.01	−0.11	0.06	Qatar	QE General	786	0.00	0.01	−0.10	0.05
Denmark	OMX Copenhagen 20	784	0.00	0.01	−0.08	0.04	Romania	BET	784	0.00	0.01	−0.12	0.07
Ecuador	Guayaquil Select	771	0.00	0.00	−0.04	0.04	Russia	MOEX	793	0.00	0.01	−0.09	0.07
France	CAC 40	805	0.00	0.01	−0.13	0.08	Saudi Arabia	Tadawul All Share	789	0.00	0.01	−0.09	0.07
Egypt	EGX 70 EWI	765	0.00	0.01	−0.08	0.05	Singapore	FTSE STS	789	0.00	0.01	−0.09	0.07
Germany	DAX	794	0.00	0.01	−0.13	0.10	Serbia	Belex 15	818	−0.00	0.01	−0.07	0.07
Greece	AGC	782	−0.00	0.02	−0.14	0.11	Slovakia	SAX	783	0.00	0.01	−0.07	0.06
Hungary	Budapest SE	777	0.00	0.01	−0.12	0.06	Slovenia	Blue–Chip SBITOP	780	0.00	0.01	−0.09	0.06
Iceland	ICEX Main	781	0.00	0.01	−0.08	0.04	South Africa	Top 40	789	0.00	0.01	−0.10	0.09
India	BSE Sensex 30	780	0.00	0.01	−0.14	0.09	Spain	IBEX 35	805	−0.00	0.01	−0.15	0.08
Indonesia	Jakarta SEC	764	−0.00	0.01	−0.07	0.10	Sri Lanka	CSE All–Share	725	0.00	0.01	−0.08	0.05
Iraq	ISX Main 60	618	0.00	0.01	−0.13	0.09	Sweden	OMX Stockholm30	789	0.00	0.01	−0.11	0.07
Ireland	ISEQ Overall	803	0.00	0.01	−0.10	0.07	Switzerland	SMI	788	0.00	0.01	−0.10	0.07
Israel	TA 35	776	0.00	0.01	−0.07	0.07	Tanzania	All Share	780	−0.00	0.01	−0.15	0.15
Italy	FTSE MIB	796	0.00	0.01	−0.18	0.08	Thailand	SET Index	769	−0.00	0.01	−0.11	0.08
Jamaica	JSE Market	741	0.00	0.01	−0.05	0.05	Tunisia	Tunindex	784	0.00	0.01	−0.04	0.03
Japan	Nikkei 225	764	0.00	0.01	−0.06	0.08	Turkey	BIST 100	790	0.00	0.01	−0.08	0.06
Kazakhstan	KASE	766	0.00	0.01	−0.05	0.03	Ukraine	PFTS	739	0.00	0.01	−0.02	0.11
Kenya	NSE20	786	−0.00	0.01	−0.05	0.03	UAE	ADX General	790	0.00	0.01	−0.08	0.08
South Korea	KOSP	774	0.00	0.01	−0.09	0.08	UK	FTSE 100	798	−0.00	0.01	−0.12	0.09
Lebanon	BLOM Stock	637	−0.00	0.01	−0.12	0.13	US	SandP 500	792	0.00	0.01	−0.13	0.09
Malaysia	FTSE KLCI	770	−0.00	0.01	−0.05	0.07	Vietnam	VN	784	0.00	0.01	−0.07	0.05
Mexico	SandP BMV IPC	791	−0.00	0.01	−0.07	0.05	Zambia	LSE All Share	778	−0.00	0.01	−0.09	0.05

*MAS, Moroccan All Shares; FTSE STS, FTSE Straits Times Singapore; AGC, Athens General Composite; UAE, United Arab Emirates; UK, United Kingdom; US, United States*.

### Results of Abnormal Returns and the COVID-19 Outbreak

Table 3 reports the summary CARs statistics over 5, 15, 30, and 60 days during the COVID-19 outbreak. Among these, the mean of CAR (0, 5), CAR (0, 15), CAR (0, 30), and CAR (0, 60) were all negative (−0.09, −0.05, −0.04, −0.03) during the outbreak. The evidence shows that the impact of a sudden disease outbreak is longer for a period and also gives investors a negative sign in terms of investment. However, a half year after the coronavirus outbreak, the stock market began to recover by itself where the mean of CAR (0,180) became positive at 0.09.

**Table 3:** Descriptive statistics of main variables.

**Variable**	**Obs**	**Mean**	**SD**	**Min**	**Max**
CAR (0,5)	68	−0.09	0.08	−0.31	0.07
CAR (0,15)	68	−0.05	0.08	−0.26	0.12
CAR (0,30)	68	−0.04	0.09	−0.28	0.19
CAR (0,60)	68	−0.03	0.09	−0.21	0.30
CAR (0,180)	68	0.06	0.16	−0.32	0.61
UHC per person	68	0.09	0.29	0.00	2.38
Total cases in 5 days	68	2,442.01	10,408.67	0.00	81,033.00
Total cases in 15 days	68	7,340.65	18,955.41	8.00	86,613.00
Total cases in 30 days	68	22,662.40	68,674.13	16.00	514,855.00
Total cases in 60 days	68	56,571.60	168,623.31	16.00	1,337,777.00
Total cases in 180 days	68	370,111.53	1,025,489.17	509.00	6,294,257.00
Log (GDP)	68	26.60	1.50	23.24	30.70
Credit/GDP	68	713.61	4,041.66	0.44	32,780.99
UAI	68	65.19	22.80	8.00	100.00
Log (Population)	68	17.10	1.53	12.77	21.05
PSNV	68	0.02	0.90	−2.56	1.53
RQ	68	3.51	1.41	0.00	5.00

*Cumulative abnormal return (CAR) is measured as the cumulative return in a country's major stock index over a period. Total cases on different days are measured as a given country's total confirmed COVID-19 cases at different periods. Universal health coverage (UHC) per person is measured as the basic health service that a person can obtain in a country. Log gross domestic product (GDP) and Credit/GDP are taken from the World Bank Open Data and represent the level of economic development. The uncertainty avoidance index (UAI) was taken from the study of Hofstede et al. (39) to control for cross-country differences in uncertainty aversion among investors. Log (Population) is taken from the World Bank Open Data and controls for the difference in the total number of residents among countries. Political stability and no violence (PSNV) and regulatory requirements (RQ) are taken from the World Bank's GOV Data 360 and indicate the quality of political safety in a country*.

The standard deviation values of CAR (0, 5), CAR (0, 15), CAR (0, 30), CAR (0, 60), and CAR (0,180) were 0.08, 0.08, 0.09, 0.09, and 0.16, respectively, where CARs (0, 180) had the highest fluctuation between positive and negative abnormal returns. The minimum and maximum values of abnormal returns were −0.32 and 0.61, showing that CARs ranged from −32 to 61% and that negative CARs will return to normal within half a year after the COVID-19 outbreak.

Table 4 illustrates the CAR results of the global stock market indices measured in different event windows. It also indicates that most CARs are significantly negative in the short term and insignificant in the long term, highlighting that COVID-19 generates negative CARs, supporting Hypothesis 1 in this study. The study results are consistent with the findings of previous studies that stock markets respond negatively to COVID-19 outbreaks because the spread of the virus encourages social distancing, causing the shutdown of financial markets. Furthermore, the high uncertainty regarding the degree of severity of the outbreak could lead to a flight to safety among investors (13, 14, 40, 41).

**Table 4:** CARs for all stock market indices.

**Country**	**Stock index**	**(1)**	**(2)**	**(3)**	**(4)**	**(5)**
		**CAR (0,5)**	**CAR (0,15)**	**CAR (0,30)**	**CAR (0,60)**	**CAR (0,180)**
ACAR	All Indices	−0.0958^***^	−0.0525^***^	−0.0379^***^	−0.0230^***^	0.0689^***^
Argentina	SandP Merval	−0.0936	0.0503	0.1580	0.2981	0.0735
Australia	SandP ASX 200	−0.1773^***^	−0.0886^***^	−0.1136^***^	−0.0188	0.0836
Austria	ATX	−0.2354^***^	−0.1190^***^	−0.1110^***^	−0.1447^**^	−0.0798
Bangladesh	DSE 30	−0.0738^***^	0.0116	0.0057	−0.0252	0.2124^**^
Belgium	BEL 20	−0.0336	−0.0017	−0.0137	−0.0665	−0.0298
Brazil	Bovespa	−0.1394^***^	−0.0997^**^	−0.0798	−0.1322	−0.1449
Bulgaria	BSE SOFIX	−0.1648^***^	−0.1447^***^	−0.1266^***^	−0.1031^**^	−0.1250
Canada	SandP TSX Composite	−0.1406^***^	−0.1115^***^	−0.0540^***^	−0.0719^**^	−0.0331
Chile	SandP CLX IPSA	−0.2392^***^	0.0071	0.0304	−0.0326	0.0024
China	Shanghai Composite	−0.0242	−0.0039	0.0025	0.0155	0.1019
Colombia	COLCAP	−0.3126^***^	−0.0798^***^	−0.1429^***^	−0.1965^***^	−0.0527
Croatia	CROBEX	−0.1838^***^	−0.0815^***^	−0.1032^***^	−0.0637^*^	−0.0068
Denmark	OMX Copenhagen 20	−0.0073	0.0798^**^	0.0863^*^	0.1047	0.1437
Ecuador	Guayaquil Select	0.0234	0.0189	0.0111	0.0195	−0.0572
France	CAC 40	−0.0488^***^	0.0081	−0.0137	−0.0701	−0.0346
Egypt	EGX 70 EWI	−0.1523^***^	−0.0871^**^	0.0652	0.1358^*^	0.5750^***^
Germany	DAX	−0.0552^***^	−0.0090	−0.0155	−0.0337	−0.0297
Greece	AGC	−0.1402^***^	0.0157	0.0445	−0.0252	0.1714
Hungary	Budapest SE	−0.1850^***^	−0.1871^***^	−0.1925^***^	−0.1640^**^	−0.1603
Iceland	ICEX Main	−0.0613^**^	0.0478	0.0756^*^	0.0678	0.2990^**^
India	BSE Sensex 30	−0.1784^***^	−0.1439^***^	−0.0397	−0.0293	0.1776
Indonesia	Jakarta SEC	−0.1587^***^	−0.0155	−0.0898^**^	−0.0418	0.1074
Iraq	ISX Main 60	−0.0206	−0.0271^**^	0.1942^***^	−0.0466^**^	0.1431
Ireland	ISEQ Overall	−0.1406^***^	−0.1175^***^	−0.1017^**^	−0.1507^**^	0.1279
Israel	TA 35	0.0083	−0.0054	−0.0181	−0.0335	−0.0885
Italy	FTSE MIB	−0.0081	−0.0018	−0.0345	−0.0807	0.0066
Jamaica	JSE Market	−0.1243^***^	−0.1481^***^	−0.2254^***^	−0.2068^***^	−0.3210^**^
Japan	Nikkei 225	−0.1119^***^	−0.1265^***^	−0.0875^*^	0.0290	0.2535
Kazakhstan	KASE	0.0121	0.0460	0.0483	0.0443	0.2316^*^
Kenya	NSE 20	−0.0966^***^	−0.0714^**^	−0.1016^**^	−0.0735	−0.0369
South Korea	KOSPI	−0.1475^***^	−0.1017^***^	−0.0806^*^	−0.0186	0.2922^**^
Lebanon	BLOM Stock	−0.0353	−0.0335	0.0294	−0.0149	0.1027
Malaysia	FTSE KLCI	−0.1449^***^	−0.0624^***^	−0.0321	0.0938^**^	0.1776^*^
Mexico	SandP BMV IPC	−0.0563^***^	−0.1418^***^	−0.1153^***^	−0.1342^**^	−0.0198
Morocco	MAS	−0.2253^***^	−0.2591^***^	−0.2747^***^	−0.2029^***^	−0.0202
Namibia	FTSE NSX Overall	−0.1492^***^	−0.1356^***^	−0.1209^**^	−0.1047	−0.0410
Netherlands	AEX	−0.0418^***^	0.0163	0.0091	−0.0181	0.0180
New Zealand	NZX 50	−0.1282^***^	−0.1028^***^	−0.0198	−0.0352	0.0175
Nigeria	NSE 30	−0.0834^***^	−0.1636^***^	−0.0662	0.1019	0.6110^***^
Norway	OSE Benchmark	−0.0232	0.0475	0.0507	0.0448	0.1360
Pakistan	Karachi 100	−0.1796^***^	−0.1615^***^	−0.1296^*^	−0.0724	0.2311
Peru	SandP Lima General	−0.1032^***^	−0.2235^***^	−0.1639^***^	−0.0768	0.0831
Philippines	PSEi Composite	−0.1585^***^	−0.1500^***^	−0.1469^***^	−0.0309	0.0925
Poland	WIG 30	0.0037	0.0906^***^	0.0624	0.0629	0.0637
Portugal	PSI 20	−0.0224	−0.0321	−0.0419	−0.0895	−0.0484
Qatar	QE General	0.0653^***^	0.1201^***^	0.1046^**^	0.1361^**^	0.0518
Romania	BET	−0.1431^***^	−0.1570^***^	−0.1533^***^	−0.0726	0.0400
Russia	MOEX	−0.0919^***^	0.0324	−0.0011	−0.0370	0.0040
Saudi Arabia	Tadawul All Share	−0.0267	0.0525	0.0233	0.0753	0.1265
Singapore	FTSE STS	−0.0267	0.0525	0.0233	0.0753	0.1265
Serbia	Belex 15	−0.1292^***^	−0.1431^***^	−0.1142^***^	−0.0853	0.0188
Slovakia	SAX	−0.0681^***^	−0.0267	0.0018	0.0914	0.0984
Slovenia	Blue-Chip SBITOP	−0.1578^***^	−0.1459^***^	−0.1058^***^	−0.0149	0.0636
South Africa	Top 40	−0.1252^***^	−0.0147	0.0160	0.0204	0.0371
Spain	IBEX 35	−0.0312	−0.0396	−0.0867^**^	−0.1258^**^	−0.0413
Sri Lanka	CSE All-Share	−0.0481^***^	−0.1137^***^	−0.1145^***^	−0.0384	0.3453^***^
Sweden	OMX Stockholm 30	0.0221	0.0243	0.0413	−0.0516	−0.0531
Switzerland	SMI	0.0191	0.0801^***^	0.0622^**^	−0.0110	−0.1439
Tanzania	All Share	−0.0798^**^	−0.1267^**^	−0.1292^*^	−0.1095	0.0805
Thailand	SET Index	−0.1620^***^	0.0002	0.0713^**^	0.1302^**^	0.1832
Tunisia	Tunindex	−0.1199^***^	−0.1415^***^	−0.1312^***^	−0.0558	0.0289
Turkey	BIST 100	−0.0640	−0.0907^*^	−0.0885	−0.0502	0.2180
Ukraine	PFTS	−0.0241	−0.0364	−0.0338	−0.0177	−0.2371
UAE	ADX General	−0.1430^***^	−0.0706^**^	−0.1088^**^	−0.1300^*^	−0.0594
UK	FTSE 100	−0.0254	−0.0341	−0.0281	−0.0398	−0.0303
US	SandP 500	0.0342^***^	0.0197^**^	0.0263^**^	0.0160	−0.0544
Vietnam	VN	−0.0879^***^	−0.0296	−0.0198	0.0798	0.3280^*^
Zambia	LSE All Share	−0.0086	−0.0013	−0.0198	−0.0412	−0.0277

*Cumulative abnormal return (CAR) is measured as the cumulative return in a country's major stock index over a period. Average cumulative abnormal return (ACAR) is measured as the average cumulative return of all country indices*.

*MAS, Moroccan All Shares; FTSE STS, FTSE Straits Times Singapore; AGC, Athens General Composite; UAE, United Arab Emirates; UK, United Kingdom; US, United States*.

^***^, ^**^, and ^*^ represent statistical significance at the 1, 5, and 10% levels, respectively.

In addition, the result of CAR (0, 60) in Table 4 verifies that the stock market recovery time is six months after the spread of COVID-19. The results appear to be the same in Bangladesh (coefficient = 0.2124; *p* < 0.05), Egypt (coefficient = 0.5750; *p* < 0.01), Iceland (coefficient = 0.2990; *p* < 0.05), South Korea (coefficient = 0.2922; *p* < 0.05), and Nigeria (coefficient = 0.6110; *p* < 0.01), where their CARs are all significant and positive. However, the Jamaican market shows the opposite result of CAR (0, 180), which is negative and significant (coefficient = −0.321; *p* < 0.05). As the Jamaican healthcare system has worsened during the COVID-19 outbreak, the government has adopted a stricter lockdown policy to prevent its spread (42, 43).

A few countries had no significant CARs during our selected data period, including Argentina (S&P Merval), Belgium (BEL 20), China (Shanghai Composite), Ecuador (Guayaquil Select), Israel (TA 35), Italy (FTSE MIB), Lebanon (BLOM Stock), Norway (OSE Benchmark), Portugal (WIG 30), Saudi Arabia (Tadawul All Share), Singapore (FTSE STS), Sweden (OMX Stockholm 30), Ukraine (PFTS), and the United Kingdom (FTSE 100). Consistent with the study by Ashraf (19), there were 14 countries in our sample that had been affected by COVID-19 before the event day of March 11, 2020. Therefore, CARs were not significant in these countries.

To test whether the spread of COVID-19 had negative abnormal returns in global stock markets, this study took the average CARs and examined whether COVID-19 led to negative abnormal returns in market indices. Our empirical results showed that COVID-19 generated negative returns for half a year after the outbreak, verifying Hypothesis 1.

### Results of the Relationship Between UHC and CARs

Table 5 reports the estimation result of Equation (3) regarding the relationship between CARs and UHC. We expected a positive and significant relationship between UHC and CARs. As shown in Table 5, the UHC per person variable was positive and significant at CAR (0, 15) (coefficient = 0.034; *p* < 0.1), CAR (0, 30) (coefficient = 0.045; *p* < 0.05), CAR (0, 60) (coefficient = 0.065; *p* < 0.01), and CAR (0,180) (coefficient = 0.188; *p* < 0.01). The exception was CAR (0, 5), which was longer than the previous period. The results have proven that CAR is positively correlated with a person who has access to adequate basic health care, consistent with Hypothesis 2. While CAR (0, 5) is not significant, the other four periods are significant and positive in relation to UHC. The empirical evidence shows that the UHC healthcare system is effective in its impact on sudden disease outbreaks in a country.

**Table 5:** Results of direct impact of UHC on cumulative abnormal returns.

**Variables**	**Cumulative abnormal return**				
	**(1)**	**(2)**	**(3)**	**(4)**	**(5)**
	**CAR (0,5)**	**CAR (0,15)**	**CAR (0,30)**	**CAR (0,60)**	**CAR (0,180)**
UHC per person	0.003	0.034^*^	0.045^**^	0.065^***^	0.188^***^
	[0.869]	[0.069]	[0.019]	[0.001]	[0.000]
Log (GDP)	0.036^***^	0.039^***^	0.048^***^	0.020^*^	0.005
	[0.002]	[0.002]	[0.001]	[0.077]	[0.781]
UAI	−0.071	0.405	0.279	0.281	0.095
	[0.856]	[0.414]	[0.579]	[0.630]	[0.921]
Credit/GDP	0.005	0.028^***^	0.025^**^	0.033^***^	0.071^***^
	[0.667]	[0.001]	[0.018]	[0.001]	[0.000]
Log (Population)	−0.033^***^	−0.033^**^	−0.042^**^	−0.007	0.031
	[0.006]	[0.016]	[0.013]	[0.584]	[0.104]
PSNV	−0.014	−0.008	−0.033	−0.000	−0.020
	[0.347]	[0.582]	[0.118]	[0.987]	[0.549]
RQ	−0.020^**^	−0.014^*^	−0.016^**^	−0.019^*^	−0.022
	[0.010]	[0.082]	[0.047]	[0.065]	[0.119]
Constant	−0.412^**^	−0.509^***^	−0.566^***^	−0.399^**^	−0.549
	[0.012]	[0.005]	[0.003]	[0.040]	[0.158]
Observations	68	68	68	68	68
Adjusted *R*^2^	0.079	0.105	0.122	0.011	0.141
*R* ^2^	0.176	0.198	0.213	0.114	0.231

*Cumulative abnormal return (CAR) is measured as the cumulative return in a country's major stock index over a period. The total number of cases on different days is measured as a given country's total number of confirmed COVID-19 cases at different periods. UHC per person is measured as the basic health service a person can obtain in a country. Log gross domestic product (GDP) and Credit/GDP are taken from the World Bank Open Data and represent the level of economic development. The uncertainty avoidance index (UAI) was taken from the study of Hofstede et al. (39) to control for cross-country differences in uncertainty aversion among investors. Log (Population) is taken from the World Bank Open Data and controls for the difference in the total number of residents among countries. Political stability and no violence (PSNV) and regulatory requirements (RQ) are taken from the World Bank's GOV Data 360 and indicate the quality of political safety in a country. P-values are given in parentheses*.

^**^, ^**^, and ^*^ represent statistical significance at the 1, 5, and 10% levels, respectively.

McKibbin and Fernando (30) indicated that countries that invest more in the public health system could reduce the negative impact of COVID-19, especially in countries with insufficient public health systems and high population density. Table 5 also shows that CARs react positively to the health system during an epidemic. The health system is a protective influence for a country in its fight against the virus and usually shows its effectiveness several days after an outbreak.

### Results of Confirmed Cases and CARs During the COVID-19 Outbreak

Table 6 presents the total confirmed cases and the interaction variable of UHC × total cases, along with other control variables, to test the moderating effects of high rates of COVID-19 infection and its connection with CARs and UHC. In this study, the total number of cases in a country is used as a proxy to denote high- and low-infected countries. The regression results in the interaction term of UHC × total cases had a negative and significant effect on CAR (0, 60) (coefficient = −0.770; *p* < 0.01) and CAR (0, 180) (coefficient = −3.367; *p* < 0.05). The results indicate that the positive impact of health coverage on CAR diminishes as the number of cases diagnosed in a country increases. The negative and significant coefficient on the interaction variable of UHC × Total cases also confirms Hypothesis 3 that the high number of diagnoses of COVID-19 may affect the impact of UHC on abnormal returns. The empirical evidence is consistent with that of Baker and Wurgler (44), Chen et al. (45), Yu and Yuan (46), and Narayan (47). They conclude that high numbers of confirmed cases will have a negative impact on markets because the increased number of cases overwhelms the primary UHC healthcare system, unevenly distributing its resources and eventually affecting the stock market in the long term.

**Table 6:** Indirect impact of UHC on CARs during the COIVD-19 pandemic.

**Variables**	**Cumulative abnormal return**				
	**(1)**	**(2)**	**(3)**	**(4)**	**(5)**
	**CAR (0,5)**	**CAR (0,15)**	**CAR (0,30)**	**CAR (0,60)**	**CAR (0,180)**
UHC per person	−0.003	0.045^**^	0.048^**^	0.050^**^	0.197^***^
	[0.886]	[0.014]	[0.031]	[0.021]	[0.000]
Total cases	0.010	0.007^*^	−0.087	−0.578	−0.496^***^
	[0.146]	[0.070]	[0.934]	[0.332]	[0.005]
UHC × Total cases	0.057	0.445	0.108	−0.770^***^	−3.367^**^
	[0.723]	[0.163]	[0.752]	[0.006]	[0.010]
Log (GDP)	0.033^***^	0.026^*^	0.046^***^	0.032^**^	0.033^*^
	[0.010]	[0.071]	[0.006]	[0.011]	[0.068]
UAI	−0.028	0.646	0.296	−0.002	0.163
	[0.944]	[0.137]	[0.565]	[0.997]	[0.852]
Credit/GDP	0.007	0.031^***^	0.024^**^	0.030^***^	0.065^***^
	[0.527]	[0.001]	[0.021]	[0.009]	[0.001]
Log (population)	−0.034^***^	−0.023^*^	−0.039^**^	−0.018	0.021
	[0.008]	[0.098]	[0.035]	[0.159]	[0.304]
PSNV	−0.017	−0.006	−0.033	0.001	−0.051
	[0.251]	[0.645]	[0.137]	[0.935]	[0.125]
RQ	−0.017^**^	−0.015^*^	−0.016^*^	−0.021^**^	−0.021
	[0.032]	[0.095]	[0.050]	[0.034]	[0.139]
Constant	−0.331^*^	−0.363	−0.570^***^	−0.494^**^	−1.100^**^
	[0.088]	[0.109]	[0.010]	[0.035]	[0.011]
Observations	68	68	68	68	68
Adjusted *R*^2^	0.066	0.118	0.093	0.046	0.262
*R* ^2^	0.191	0.236	0.215	0.175	0.362

*Cumulative abnormal return (CAR) is measured as the cumulative return in a country's major stock index over a period. The total number of cases on different days is measured as a given country's total number of confirmed COVID-19 cases at different periods. UHC per person is measured as the basic health service a person can obtain in a country. Log gross domestic product (GDP) and Credit/GDP are taken from the World Bank Open Data and represent the level of economic development. The uncertainty avoidance index (UAI) was taken from the study of Hofstede et al. (39) to control for cross-country differences in uncertainty aversion among investors. Log (Population) is taken from the World Bank Open Data and controls for the difference in the total number of residents among countries. Political stability and no violence (PSNV) and regulatory requirements (RQ) are taken from the World Bank's GOV Data 360 and indicate the quality of political safety in a country. P-values are given in parentheses*.

^***^, ^**^, and ^*^ represent statistical significance at the 1, 5, and 10% levels, respectively.

## Conclusion

To the best of our knowledge, this is the first study that adopted the healthcare system to evaluate its effect during the COVID-19 outbreak on the stock market. The ongoing COVID-19 pandemic has attracted attention in every country with regard to its impact on people's daily lives. The UHC healthcare system enables everyone to avoid risk exposure, including prevention and treatment in the COVID-19 crisis period. In particular, a significant shock in the stock market is often triggered by an event that significantly reduces trust in investors in a security market because major global events or crises have impacted the global economy and financial markets (48). Our findings addressed the important points of the correlation between healthcare system and stock prices' movement by showing how a country's UHC responds to a sudden disease outbreak through abnormal returns and makes it more susceptible to citations from the academic literature.

The empirical results of this study reveal that each country's stock index had significantly negative abnormal returns in the short term but not in the long term. Thus, the CAR reacted significantly at the beginning of the pandemic. This is because people may lose confidence or panic because of the shock of the sudden outbreak of a pandemic, incurring negative investor sentiment in the stock market. However, once the UHC is working optimally, the negative reaction to the stock market will disappear in the long term. We also examined the direct and indirect impact of health coverage on CARs during the pandemic. To this end, we ran an OLS regression to calculate the correlation between UHC and CARs on the event days of 5, 10, 30, 60, and 180. The results showed that the effectiveness of UHC remained positive and strongly significant after a period of the COVID-19 outbreak. Nevertheless, the impact was reversed in countries with a higher number of confirmed cases. In reality, when the healthcare system can no longer fulfill the needs of society or individuals to maintain their daily life, chaos and panic ensue due to insufficient or limited resources.

The findings of this study provide several perspectives on financial markets. CARs emerge at the early stages of the pandemic, signifying that the strategy of investment as a sudden reaction to the outbreak is normally at the beginning of the pandemic and that a well-organized UHC system is a key factor in avoiding the risk of damage to stock markets as a result of a sudden outbreak. Further, the results also show that UHC can gradually reduce CARs in the long term, whereas the COVID-19 outbreak has a negative impact on stock markets in the short term.

Although our analysis reveals some important insights into the correlation of UHC and abnormal returns in a short-term response during a pandemic disease, it disregards the effects of lockdown, vaccination coverage in a population, its connection with stock markets in a long-term reaction, and the impact on socio-economics as well. This limitation can be addressed in future studies to assess various measures used to limit the spread of COVID-19, and relieve the pressure on health care systems, travel, consumption and investment, and logistics, causing so-called socio-economic impacts at the market.

## Data Availability Statement

The original contributions presented in the study are included in the article/supplementary material, further inquiries can be directed to the corresponding author.

## Author Contributions

C-HT contribution is reflected in the choice of specialized literature, the definition of research hypotheses, investigation, writing-original draft preparation, visualization, and editing. Y-HL contribution is reflected in the definition of the sample, testing of hypotheses, statistical data processing, resources, and discussion. WL contribution is reflected in the data collection, formal analysis, and conclusions. LW helps to organize the literature review. Y-HL and WL contributed in interpretation of results. All authors have read and agreed to the published version of the manuscript.

## Funding

The authors acknowledge this study was supported by Guangxi First Class Discipline-the Statistics construction project fund. This study was also supported by the Center of Econometric Application in Accounting and Finance.

## Conflict of Interest

The authors declare that the research was conducted in the absence of any commercial or financial relationships that could be construed as a potential conflict of interest.

## Publisher's Note

All claims expressed in this article are solely those of the authors and do not necessarily represent those of their affiliated organizations, or those of the publisher, the editors and the reviewers. Any product that may be evaluated in this article, or claim that may be made by its manufacturer, is not guaranteed or endorsed by the publisher.

## Footnotes

^1^This study did not include indices from countries whose data were not fully available or whose data were not completely publicly available (e.g., a country that is not a member of the WHO, such as Taiwan, Bahrain, Uganda, etc.).

^2^Universal Health Coverage Index encompasses two values: SDG indicator 3.8.1 and SDG indicator 3.8.2. However, WHO (2) only gave the formula for the first indicator; therefore, the calculation of UHC was not revealed entirely.
